# A simple and efficient method to enhance audiovisual binding tendencies

**DOI:** 10.7717/peerj.3143

**Published:** 2017-04-25

**Authors:** Brian Odegaard, David R. Wozny, Ladan Shams

**Affiliations:** 1 Department of Psychology, University of California, Los Angeles, Los Angeles, CA, United States; 2 Department of Bioengineering, University of California, Los Angeles, Los Angeles, CA, United States; 3 Neuroscience Interdepartmental Program, University of California-Los Angeles, Los Angeles, CA, United States

**Keywords:** Binding tendency, Sensory integration, Multisensory integration, Multisensory learning, Multisensory plasticity, Bayesian causal inference

## Abstract

Individuals vary in their tendency to bind signals from multiple senses. For the same set of sights and sounds, one individual may frequently integrate multisensory signals and experience a unified percept, whereas another individual may rarely bind them and often experience two distinct sensations. Thus, while this binding/integration tendency is specific to each individual, it is not clear how plastic this tendency is in adulthood, and how sensory experiences may cause it to change. Here, we conducted an exploratory investigation which provides evidence that (1) the brain’s tendency to bind in spatial perception is plastic, (2) that it can change following brief exposure to simple audiovisual stimuli, and (3) that exposure to temporally synchronous, spatially discrepant stimuli provides the most effective method to modify it. These results can inform current theories about how the brain updates its internal model of the surrounding sensory world, as well as future investigations seeking to increase integration tendencies.

## Introduction

In many studies investigating human perception of concurrent visual and auditory stimuli, individuals exhibit different degrees of multisensory integration; for the same set of stimuli, some individuals integrate often, while others do not integrate at all (Mcgurk & Macdonald, 1976; Hairston et al., 2003; Tremblay et al., 2007a; Stevenson, Zemtsov & Wallace, 2012; Nath & Beauchamp, 2012; Hass et al., 2012; Magnotti & Beauchamp, 2014; Mallick, Magnotti & Beauchamp, 2015; Gurler et al., 2015). Recently, we have shown that this variability across individuals in sensory tasks is at least partially due to differences in the *tendency to bind* crossmodal signals (Odegaard & Shams, 2016). However, little is currently known about how experience may contribute to an individual’s binding tendency in a given task. Additionally, while many studies have shown that interactions between the senses change over the course of development (Neil et al., 2006; Tremblay et al., 2007b; Gori et al., 2008; Barutchu, Crewther & Crewther, 2009; Barutchu et al., 2010; Hillock, Powers & Wallace, 2011; Hillock-Dunn, Grantham & Wallace, 2016), the degree to which an individual’s binding tendency remains plastic in adulthood is unknown.

Therefore, we conducted the following investigation to explore two questions: (1) can brief sensory experiences alter the brain’s tendency to bind audiovisual signals in adulthood; (2) If so, what are the spatial and temporal relationships between visual and auditory stimuli that cause it to change? In pursuit of answers to these questions, we investigated the plasticity of the binding tendency in the context of audiovisual spatial perception. Spatial perception is a fundamental brain function involved in many tasks that the brain has to perform frequently, and sophisticated computational tools have been developed that can be utilized for rigorously and quantitatively measuring both unisensory processing and the binding tendency in spatial tasks (Körding et al., 2007; Wozny, Beierholm & Shams, 2010; Odegaard, Wozny & Shams, 2015). Specifically, human performance in audiovisual spatial tasks is modeled quite effectively by Bayesian causal inference, where an observer’s perception of simultaneous audiovisual signals depends not only on the reliability of unisensory encoding (modeled by sensory likelihoods), but also upon the inference of whether a common cause is the source of both the auditory and visual signals (which is influenced by a prior bias for binding the senses) (Körding et al., 2007; Beierholm, Quartz & Shams, 2009; Wozny, Beierholm & Shams, 2010; Rohe & Noppeney, 2015a; Odegaard, Wozny & Shams, 2015; McGovern et al., 2016; Odegaard, Wozny & Shams, 2016; Odegaard & Shams, 2016). This prior governing integration and segregation, referred to as the “binding tendency” (Odegaard, Wozny & Shams, 2016; Odegaard & Shams, 2016) is quantifiable, stable over time, and provides a true measure of the brain’s capacity to bind audiovisual information which is not confounded by unisensory encoding (Odegaard & Shams, 2016); however, it remains an open question of how quickly and under what conditions it may change.

Previous research has indicated that some forms of audiovisual spatial processing are highly plastic and update on a scale of milliseconds (Wozny & Shams, 2011a), with these changes best accounted for by shifts in sensory likelihoods (Wozny & Shams, 2011b). As sensory spatial processing appears to be modifiable in adulthood by short-term sensory experience (Chen & Vroomen, 2013), we investigated whether the binding tendency could also be modified by brief exposures to audiovisual stimuli.

**Figure 1:**
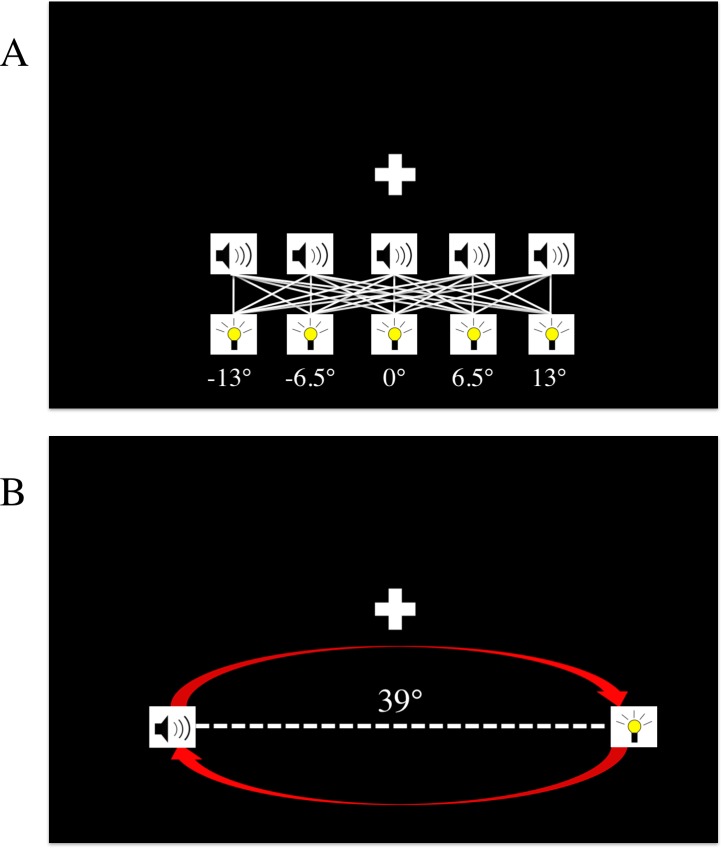
The spatial localization paradigm and exposure tasks. (A) The localization task. Stimuli could be presented from one of five locations, ranging from −13 to +13 degrees. On any given trial, subjects could be presented with only an auditory stimulus, only a visual stimulus, or both an auditory and visual stimulus. The white lines in the figure designate all of the different spatial combinations of the bisensory trials, and the white “+” symbol denotes the fixation cross. (B) The exposure task paradigm. This was interleaved with localization blocks in the post-test phase. Depicted above is the stimulus arrangement from experiments #2, #5, and #6 where auditory and visual stimuli were always presented from 39 degrees apart, and alternated positions with each correct detection of the brighter flash. In experiments #3 and #4, the auditory and visual stimuli always were presented from the same location (−19.5 or +19.5 degrees), and alternated together from one side to the other. In experiment #1, no exposure task was implemented, and subjects simply listened to music during blocks when the normal exposure task occurred in other experiments.

To explore this question, we conducted six different experiments to gain insights into the conditions that could cause an update of this prior. In all experiments, the basic design was as follows: first, the binding tendency for each observer was quantified by having subjects perform a spatial localization task with a variety of stimulus conditions, which included both unisensory (auditory or visual) and bisensory (simultaneous audiovisual) stimuli (Fig. 1A). Next, the observers were presented with a brief “exposure phase” in which they were passively exposed to stimuli with various spatial and temporal relationships. Figure 1B shows an example arrangement of stimuli during the exposure phase, and Table 1 shows how the temporal and spatial relationships between the auditory and visual stimuli in the exposure phase varied across the six experiments (but see Materials and Methods for additional details).

**Table 1:** Spatial and temporal relationships of exposure-task stimuli in the six experiments. The “time” column denotes whether the stimuli in the exposure task were temporally congruent (i.e., synchronous), uncorrelated (with timing of the auditory stimulus drawn from a uniform distribution of −250 ms to +250 ms of when the visual stimulus occurred) or correlated (always separated by a fixed, 400 ms offset, with the visual stimulus preceding the auditory stimulus). The “space” column refers to whether exposure phase stimuli were incongruent (presented from 39 degrees apart, alternating positions with each correct detection of the brighter flash), or congruent (presented from the same location, alternating sides with each correct detection of the brighter flash).

Experiment	Time	Space
1	None	None
2	Uncorrelated	Incongruent
3	Congruent	Congruent
4	Uncorrelated	Congruent
5	Congruent	Incongruent
6	Correlated	Incongruent

The main part of the exposure phase lasted approximately ten minutes. Finally, the participants completed the same localization task as the one prior to exposure, to quantify any changes in the binding tendency following the exposure phase. To avoid having the effects of the main exposure phase wash out over this period, we employed brief refresher blocks of the exposure task in a “top-up” design every 40 trials in the post-test localization session. By comparing differences in the binding tendency in the localization task between the pre-test and post-test phases, we could examine whether any of the exposures caused the brain to update its tendency to bind spatially discrepant audiovisual signals in the post-test localization phase.

In our simple spatial localization task, two dimensions are relevant in the brain’s inference of whether or not signals should be integrated or segregated: *space*, and *time*. In each experiment’s exposure phase, subjects passively observed audiovisual stimuli with various spatial and temporal relationships. Given that brief flashes and sounds that are presented simultaneously and at the same location are generally perceived to have a common cause (Wallace et al., 2004; Rohe & Noppeney, 2015b), repeated exposure to spatio-temporally congruent flashes could potentially serve as a cue to update the binding tendency. Similarly, given that flashes and sounds with a large spatial discrepancy or temporal discrepancy are often perceived as originating from independent sources, this could also serve as a cue to update the binding tendency (Wallace et al., 2004; Rohe & Noppeney, 2015b). While it is not immediately clear *how* these cues would serve to update the binding tendency, as the conditions necessary to cause an update in multisensory priors are currently unknown, general ideas from sensory research motivated our two hypotheses about when sensory experience may cause our internal models of the world to change.

**Figure 2:**
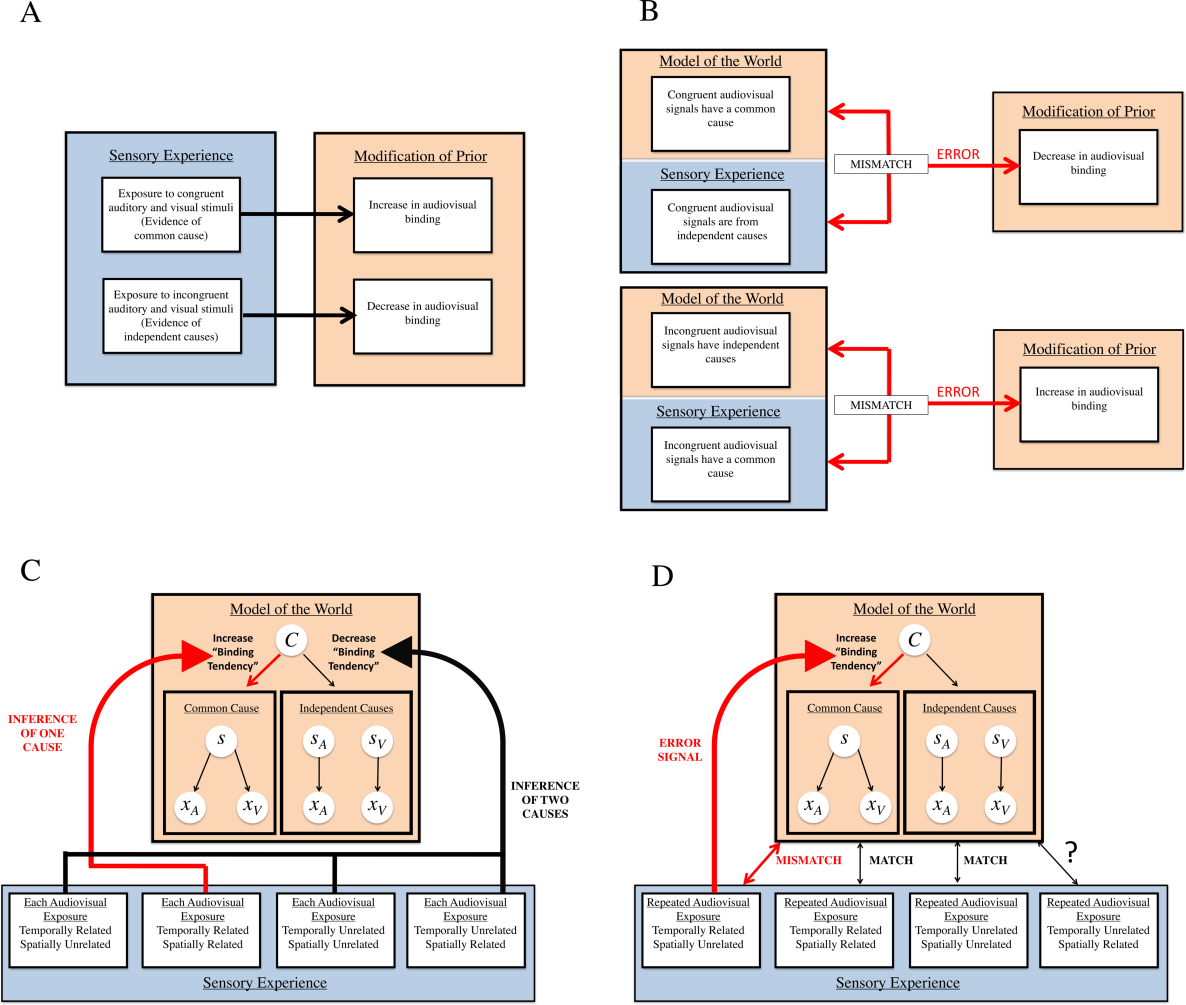
Our two hypotheses of how the binding tendency may change: general predictions, and specific applications to Bayesian causal inference. (A) Hypothesis #1, general predictions: priors are updated to reflect statistics of stimuli. Within our first hypothesis, all types of sensory experience are informative of relationships between auditory and visual signals in the environment, and therefore should cause a change in audiovisual binding. For instance, exposure to audiovisual stimuli that are *congruent* (either in space or in time) should lead to an increase in audiovisual binding, as the brain learns the association between the stimuli and updates its model of the world. Similarly, exposure to *incongruent* audiovisual stimuli should lead to a decrease in audiovisual binding. (B) Hypothesis #2, general predictions: mismatch between internal model and sensory experience requires modification. Within our second hypothesis, *mismatches* between the current model of the world and sensory experience are most informative regarding novel relationships between auditory and visual signals that the brain must take into account. Thus, whenever sensory experience indicates that congruent audiovisual stimuli actually originate from independent causes, this should change the brain’s internal model to decrease audiovisual binding. Similarly, if experience indicates that incongruent audiovisual stimuli actually originate from a common cause, the brain should increase binding of audiovisual information. (C) Hypothesis #1 and Bayesian causal inference. In the Bayesian causal inference framework, according to the first hypothesis, each audiovisual exposure serves to update the prior for binding (i.e., binding tendency). Evidence of a common cause by each exposure to congruent signals accumulates in this prior, increasing the tendency to bind, and evidence of independent causes by each exposure to incongruent signals decreases the tendency to bind. (D) Hypothesis #2 and Bayesian causal inference. According to our second hypothesis, the brain may only update its internal model of the world when there is a mismatch between sensory evidence and the current model. In our experiments, only conditions which present repeated evidence (i.e., strong temporal relationships between audiovisual stimuli) that signals thought to originally have independent causes actually originate from a common cause produce an error signal, and thus require an update of the internal model of the world. As evidence indicates that spatial relationships are not as strong of a cue as temporal relationships in updating integration tendencies (Wallace & Stein, 2007), it is unlikely that repeated presentation of temporally unrelated and spatially related stimuli would be potent enough to modify the brain’s tendency to bind.

First, research on statistical learning in human observers has shown that exposure to sensory stimuli with statistical regularities (e.g., joint probabilities, conditional probabilities) causes learning of the statistical regularities (Saffran, Aslin & Newport, 1996; Fiser & Aslin, 2016; Fiser & Aslin, 2002). If the binding tendency, which is equivalent to the *a priori* probability of a common cause, is indeed plastic and can be updated by brief experience, it may change to ultimately mirror the statistics of the environment. Therefore, it seems possible that repeated exposure to sounds and sights that are congruent in the relevant dimensions for sensory inference in this task (space and time) would indicate these signals come from a common cause, and should therefore result in an *increase* in the binding tendency (Parise, Spence & Ernst, 2012). Conversely, repeated exposure to sounds and sights that are incongruent in a given dimension would indicate these signals come from separate sources, and should result in a *decrease* in the binding tendency (Fig. 2A).

However, another prominent idea in sensory perception research, predictive coding, presents an alternative idea about when the brain may update priors that influence perception of the world. One of the main tenets of predictive coding is that mismatches between what the brain predicts and actual sensory experience leads to an updating of the brain’s internal model of the world (Rao & Ballard, 1999; Friston, 2003; Friston, 2012; Talsma, 2015). According to its internal model of the world, the brain would not predict that sensory signals that are incongruent in a given dimension would have a common cause and would repeatedly co-occur. However, when repeatedly presented with temporally coincident but otherwise discrepant stimuli, enough evidence is provided to the brain to signal an association between the stimuli (i.e., a common cause), and this mismatch between the expectation/prediction and sensory experience produces an error signal. According to this idea, the nervous system would modify its internal model of the world to allow for these discrepant signals to be perceived as unified (Fig. 2B).

Thus, interestingly, these ideas lead to different predictions about when the binding tendency may get updated as a result of our exposure tasks. For example, shown in Fig. 2C is our first hypothesis. In this framework, the internal model of the world is a generative model of how sensory signals are produced by causes in the outside world. For any two sensory signals (*x*_*A*_ and *x*_*V*_), they are either caused by the same source (*s*), or by two independent sources (*s*_*A*_ and *s*_*V*_). The prior probability of the common cause hypothesis is the “binding tendency,” which is the *a priori* bias of the nervous system to expect a common cause for any two sensory (here, audiovisual) signals. In Fig. 2C, the light blue box depicts four (out of many) possible types of sensory experience, and these correspond to the four types of exposures used in the current study across the six experiments. In this framework, the statistics of sensory stimuli with a common cause would lead to an increase of prior for the common cause (or binding tendency), and vice versa. On each exposure-phase stimulus presentation where the audiovisual stimulus leads to inference of a common cause (because they are spatiotemporally congruent), it would slightly increase the binding tendency (shown by the red arrow). On each exposure presentation where the audiovisual stimulus leads to an inference of two causes (because the audiovisual stimuli are too discrepant in either space or time), it would slightly decrease the binding tendency (shown by the black arrow). Over the course of many trials, these changes would accumulate. The change in the binding tendency would occur on every single trial, but the change would accumulate across trials and become more evident as more experience is acquired.

However, our second hypothesis (as shown in Fig. 2D) makes different predictions about when changes should occur to the binding tendency. For example, neurophysiological studies provide evidence that *time* may be a more important factor than *space* in driving plasticity of multisensory integration capacities, and that strong temporal associations between audiovisual signals can cause an increase in the capacity to integrate spatially discrepant stimuli (Wallace & Stein, 2007). Therefore, in our experiments, only one type of sensory experience (which was used in Experiment 5 and Experiment 6), wherein the auditory and visual signals are presented with strong temporal consistency and spatial inconsistency, would violate the predictions of the internal model. Repeated temporal coincidence of audiovisual signals can provide strong evidence of unity, which can be at odds with large spatial discrepancies. Because of the mismatch between the model prediction and the sensory data over repeated trials, an error signal is produced, which is then used to modify the internal model by adjusting the binding tendency. This process is shown by the red arrow. By increasing the binding tendency, the nervous system becomes more tolerant towards spatial discrepancies between the two sensory signals in perceiving unity. Alternatively, signals that are either fully related or unrelated do not produce any mismatch with repeated presentation, and thus an update of the internal model of the world is not warranted.

## Materials and Methods

### Participants

A total of 189 subjects (ages 18–55) participated in this study. Twenty-one subjects were excluded due to either negligence with the response device or eyetracker malfunctions during the task. Thus, analyses for each experiment included between 22–31 subjects. A subset of the “pre-test” data in these experiments was used in a previous paper to compare the performance of various Bayesian models to account for multisensory spatial localization abilities (Odegaard, Wozny & Shams, 2015). All participants verbally reported that they did not have a history of any neurological conditions (seizures, epilepsy, stroke), had normal or corrected-to-normal vision and hearing, and had not experienced head trauma. The University of California North General IRB granted ethical approval to carry out the study in our lab (IRB#13-000476), and each subject provided written consent on the consent form approved by the UCLA IRB.

### Apparatus and stimuli

Eligible participants sat at a desk in a dimly lit room with their chins positioned on a chinrest 52 cm from a screen. The screen was a black, acoustically transparent cloth subtending much of the visual field (134° width° × 60° height). Behind the screen were seven free-field speakers (5 × 8 cm, extended range paper cone), positioned along azimuth 6.5° apart, 7° below where the fixation cross was displayed. The middle speaker was positioned below the fixation point, and three speakers were positioned to the right and three to the left of fixation.

The visual stimuli were presented overhead from a ceiling mounted projector set to a resolution of 1,280 × 1,024 pixels with a refresh rate of 75 Hz. The visual stimuli used in the experiments were white-noise disks (.41 cd/m2) with a Gaussian envelope of 1.5° FWHM, presented 7° below the fixation point on a black background (.07 cd/m2), for 35 ms. The center of visual stimuli overlapped the center of one of the five speakers behind the screen positioned at −13°, −6.5°, 0°, 6.5°, and 13°. Auditory stimuli were ramped white noise bursts of 35 ms measuring 59 dB(A) sound pressure level at a distance of 52 cm. The speaker locations were unknown to all participants in the experiment.

Prior to the presentation of any stimuli in the experiment, participants were required to have their gaze centered on a central fixation point, which was a small, white cross. To ensure that participants’ gaze for each trial was starting from the same location, gaze position and fixation time were recorded at 60 Hz with a ViewPoint eye tracker (Arrington Research, Scottsdale, AZ, USA) fixed to the chinrest and PC-60 software (version 2.8.5,000). Stimuli were not displayed until the recorded gaze angle was within 3.0° of the fixation point and the fixation time was greater than 250 ms. Viewing of the stimuli was binocular, although only movements of the right eye were tracked. The eye tracker was adjusted for each participant before the test session to ensure that the entire eye was being monitored, and a calibration task was performed before trials for the experiment began. A separate computer controlled stimuli presentation and recorded behavioral responses using MATLAB (version 7.6.0, R2008a). A wireless mouse was used to record the behavioral responses.

### Bayesian causal inference model

Following collection of each individual subject’s data, responses were divided into the pre-test and post-test datasets, and a Bayesian Causal Inference model with 8 free parameters (Odegaard, Wozny & Shams, 2015; Odegaard, Wozny & Shams, 2016) was fit to the data (see Supplemental Information for further details). The free parameters included the “binding tendency” (*b*_*t*_), which in this Bayesian framework is the prior probability of inferring a common cause, a prior distribution over space characterized by a mean (*x*_*p*_) and variance (*σ*_*p*_), and five parameters characterizing the sensory likelihood distributions. These likelihood parameters included the standard deviation of the visual likelihood (*σ*_*v*_), how the visual likelihood variance scales with eccentricity (Δ*σ*_*v*_), how bias in the visual likelihood mean scales with eccentricity (Δ*x*_*v*_), the standard deviation of the auditory likelihood distributions (*σ*_*a*_), and how bias in the auditory likelihood mean scales with eccentricity (Δ*x*_*a*_).

We included these likelihood parameters to determine whether any changes in participants’ localization behaviors were due to a change in *binding tendency* (i.e., the prior probability of inferring a common cause, or “*p*_common_”), or a change in the sensory representations of space. Previously, it has been well established that repeated exposure to spatially discrepant audiovisual stimuli can result in subsequent auditory localizations being mislocalized towards the direction of the previous visual stimulus (Canon, 1970; Radeau & Bertelson, 1977; Recanzone, 1998; Lewald, 2002; Frissen et al., 2003; Frissen et al., 2005; Bertelson et al., 2006; Frissen, Vroomen & De Gelder, 2012), and that this effect can be accounted for by a shift in the auditory likelihoods (Sato, Toyoizumi & Aihara, 2007; Wozny & Shams, 2011b). In order to allow for this possible explanation of the data, we needed to include these additional parameters to determine whether any potential changes induced by the exposure task were due to changes in *sensory representations*, or changes in the *binding tendency*. In addition to the aforementioned parameters, the perceptual decision-making strategy (probability matching strategy, model averaging strategy, or model selection strategy (Wozny, Beierholm & Shams, 2010)) was also fitted to the data for each individual participant, and parameters from the best-fitting strategy were analyzed.

**Figure 3:**
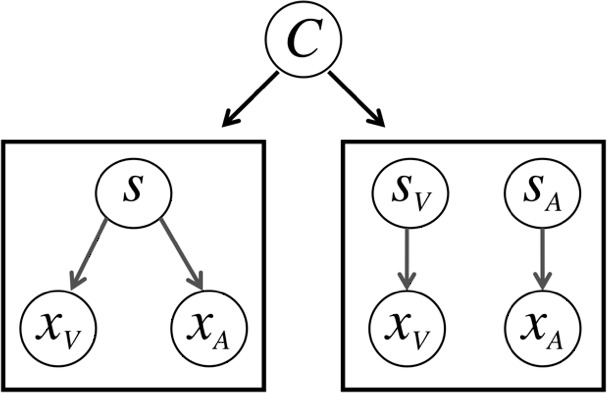
The causal inference model. On some trials, the observer may infer that both the auditory (*x*_*A*_) and visual (*x*_*V*_) signals come form a single source (*s*) and should be integrated. On other trials, the observer may infer that the signals come from two sources (*s*_*V*_ and *s*_*A*_) and should be segregated. The latent variable *C* determines which model generates the data.

Shown in Fig. 3, the general idea behind the model is that in this task, observers only have access to sensory signals *x*_*A*_ and *x*_*V*_, and must determine whether or not the signals come from one source and should be integrated, or come from two sources and should be segregated.

The noisy sensory signals that the subjects have access to are modeled by likelihood distributions for the visual and auditory modalities. The means for these likelihood distributions (*x*_*A*_ *and x*_*V*_) are sampled from a normal distribution around the true location of the stimulus (*s*_*A*_ *and s*_*V*_) to simulate the corruption of sensory channels by the noise parameters *σ*_*V*_ and *σ*_*A*_, which capture the inherent precision of encoding in each sensory modality. A delta term is also included to capture biases in the two sensory channels’ representations. This delta terms scales with eccentricity, based on the spatial position *K* of each stimulus; each value of *K* corresponds to a specific spatial position (i.e., −13° corresponds to −2, −6.5° corresponds to −1, 0° corresponds to 0, 6.5° corresponds to 1, and 13° corresponds to 2). If the delta term is negative, this will account for central biases in the sensory representations; if the delta term is positive, this will account for peripheral biases in the sensory representations. We also include a delta term for the visual likelihood noise that scales with stimulus eccentricity, as shown in the equations below:

(1) \begin{eqnarray*}& & {x}_{V}\sim N({s}_{V}+(\Delta {x}_{V}K),{\sigma }_{V}+{|}\Delta {\sigma }_{V}K{|})\end{eqnarray*}

\begin{eqnarray*}& & K=\{-2,-1,0,1,2\} \end{eqnarray*}

(2) \begin{eqnarray*}& & {x}_{A}\sim N({s}_{A}+(\Delta {x}_{A}K),{\sigma }_{A})\end{eqnarray*}

\begin{eqnarray*}& & K=\{-2,-1,0,1,2\}. \end{eqnarray*}

In this model, observers’ final estimates are not only influenced by noise in the sensory likelihoods and biases in how those distributions are represented, but also by pre-existing *a priori* biases over space. Thus, we also include a spatial prior, as this element has been shown to significantly improve the model fits.

(3) \begin{eqnarray*}p(s)=N({\mu }_{p},{\sigma }_{P})\end{eqnarray*}

The likelihoods and spatial prior are combined to compute the posterior probability of a sensory event. The more similar the sensory signals are, the more likely scenario is that they have originated from the same source and thus should be integrated; with more discrepant sensory signals, it is more likely they originated from separate sensory signals and thus should be segregated. Thus, the posterior probability of a sensory event *s* is conditioned on the causal structure of the stimuli, with the optimal combinations according to each causal scenario listed below:

(4) \begin{eqnarray*}& & p(s{|}{x}_{A},{x}_{V};C=1)= \frac{p({x}_{A}{|}s)p({x}_{V}{|}s)p(s)}{p({x}_{A},{x}_{V})} \end{eqnarray*}

(5) \begin{eqnarray*}& & p({s}_{A}{|}{x}_{A};C=2)= \frac{p({x}_{A}{|}{s}_{A})p(s)}{p({x}_{A})} \end{eqnarray*}

(6) \begin{eqnarray*}& & p({s}_{V}{|}{x}_{V};C=2)= \frac{p({x}_{V}{|}{s}_{V})p(s)}{p({x}_{V})} \end{eqnarray*}

The maximum a posteriori estimate of these computed posteriors reflects the optimal combination of the sensory signals given one or two causes. (Note that Eqs. (5) and (6) are also used to model the unisensory visual and unisensory auditory data.) Since subjects do not know whether or not the signals were generated by one or two sources, this must be inferred, incorporating both the sensory likelihoods and the prior information as well. This process of inference can be computed according to Bayes’ Rule:

(7) \begin{eqnarray*}p(C{|}{x}_{A},{x}_{V})= \frac{p({x}_{A},{x}_{V}{|}C)p(C)}{p({x}_{A},{x}_{V})} \end{eqnarray*}

In order to compute the posterior probability of a single cause, this can be done in the following manner, with *p*_*c*_ denoting the prior probability of the common cause. This prior probability of a common cause, *p*_*c*_, is the measure of interest in this manuscript (i.e., it is the binding tendency). Values near 1 indicate that nearly all bisensory stimuli, regardless of spatial discrepancy, will be integrated, while values near 0 indicate that nearly all bisensory stimuli will be segregated:

(8) \begin{eqnarray*}p(C=1{|}{x}_{A},{x}_{v})= \frac{p({x}_{A},{x}_{V}{|}C=1){p}_{c}}{p({x}_{A},{x}_{V}{|}C=1){p}_{c}+p({x}_{A},{x}_{V}{|}C=2)(1-{p}_{c})} \end{eqnarray*}

In this equation, the likelihood terms can be computed by integrating over the latent variable *s*:

(9) \begin{eqnarray*}& & p({x}_{A},{x}_{V}{|}C=1)=\int \nolimits p({x}_{A}{|}s)p({x}_{V}{|}s)p(s)ds\end{eqnarray*}

(10) \begin{eqnarray*}& & p({x}_{A},{x}_{V}{|}C=2)=\int \nolimits p({x}_{A}{|}{s}_{A})p({s}_{A})d{s}_{A}\cdot \int \nolimits p({x}_{V}{|}{s}_{V})p({s}_{V})d{s}_{V}\end{eqnarray*}

Following integration over the latent variable *s*, the posterior probability of a common cause can be computed, and thus, the posterior probability of two causes can also be computed:

(11) \begin{eqnarray*}p(C=2{|}{x}_{A},{x}_{V})=1-p(C=1{|}{x}_{A},{x}_{V}).\end{eqnarray*}

Finally, it must be determined how this inferred posterior probability can be used to weight the various sensory estimates. Three possible perceptual strategies exist, as shown in the following section:

Model Selection:

(12) \begin{eqnarray*}\begin{array}{@{}l@{}} \displaystyle {\hat {s}}_{A}= \left\{ \begin{array}{@{}l@{}} \displaystyle {\hat {s}}_{A,c=1}~\text{if}~p(C=1{|}{x}_{A},{x}_{V})\gt .5 \\ \displaystyle {\hat {s}}_{A,c=2}~\text{if}~p(C=1{|}{x}_{A},{x}_{V})\leq .5 \end{array} \right. \\ \displaystyle {\hat {s}}_{V}= \left\{ \begin{array}{@{}l@{}} \displaystyle {\hat {s}}_{v,c=1}~\text{if}~p(C=1{|}{x}_{A},{x}_{V})\gt .5 \\ \displaystyle {\hat {s}}_{V,c=2}~\text{if}~p(C=1{|}{x}_{A},{x}_{V})\leq .5 \end{array} \right. \end{array}\end{eqnarray*}

Model Averaging:

(13) \begin{eqnarray*}\begin{array}{@{}l@{}} \displaystyle {\hat {s}}_{A}=p(C=1{|}{x}_{A},{x}_{V}){\hat {s}}_{A,C=1}+p(C=2{|}{x}_{A},{x}_{V}){\hat {s}}_{A,C=2}\\ \displaystyle {\hat {s}}_{V}=p(C=1{|}{x}_{A},{x}_{V}){\hat {s}}_{V,C=1}+p(C=2{|}{x}_{A},{x}_{V}){\hat {s}}_{V,C=2} \end{array}\end{eqnarray*}

Probability Matching:

(14) \begin{eqnarray*}\begin{array}{@{}l@{}} \displaystyle {\hat {s}}_{A}= \left\{ \begin{array}{@{}l@{}} \displaystyle {\hat {s}}_{A,c=1}~\text{if}~p(C=1{|}{x}_{A},{x}_{V})\gt \xi \\ \displaystyle \text{where}~\xi \in [0:1]~\text{uniform distribution} \\ \displaystyle {\hat {s}}_{A,c=2}~\text{if}~p(C=1{|}{x}_{A},{x}_{V})\leq \xi \\ \displaystyle \text{and sampled on each trial} \end{array} \right. \\ \displaystyle {\hat {s}}_{V}= \left\{ \begin{array}{@{}l@{}} \displaystyle {\hat {s}}_{V,c=1}~\text{if}~p(C=1{|}{x}_{A},{x}_{V})\gt \xi \\ \displaystyle \text{where}~\xi \in [0:1]~\text{uniform distribution} \\ \displaystyle {\hat {s}}_{V,c=2}~\text{if}~p(C=1{|}{x}_{A},{x}_{V})\leq \xi \\ \displaystyle \text{and sampled on each trial}. \end{array} \right. \end{array}\end{eqnarray*}

For each subject, we fit eight free parameters to individual subjects’ data, and also determined whether subjects data were best classified by a probability matching strategy, model averaging strategy, or model selection strategy (Wozny, Beierholm & Shams, 2010, see study #1 for details). This was done simulating 10,000 trials using MATLAB’s *fminsearchbnd* function (Mathworks, 2009), maximizing the likelihood of the parameters of the model. The parameter fits from the best-fitting strategy for each experiment are shown in the tables in the Supplemental Information.

### Procedure

Participants began each session by being exposed to 10 practice trials with only unimodal auditory stimuli presented. This practice session ensured that participants were accurately using the mouse, understood the instructions, and were accurately fulfilling the fixation requirements for each trial. Each trial started with the fixation cross, followed after 750 ms (if the subject was fixating properly) by the presentation of stimuli. 450 ms after the stimuli, fixation was removed and a cursor appeared on the screen just above the horizontal line where the stimuli were presented, and at a random horizontal location in order to minimize response bias. The cursor was controlled by the trackball mouse placed in front of the subject, and could only be moved in the horizontal direction (i.e., to the left and right). Participants were instructed to “move the cursor as quickly and accurately as possible to the exact location of the stimulus and click the mouse.” This enabled the capture of continuous responses with a resolution of 0.1 degree/pixel. No feedback about the correctness of responses was given. Participants were allowed to move their eyes as they made their response.

Following the brief practice session, participants began the “pre-test” session, which consisted of 525 trials of interleaved auditory, visual, and audiovisual stimuli presented in pseudorandom order, which took about 45 min to complete, including break screens that would arise approximately every 9–10 min. The stimulus conditions included five unisensory auditory locations, five unisensory visual locations, and 25 combinations of auditory and visual locations (bisensory conditions), for a total of 35 stimulus conditions (15 trials per condition). In the bisensory conditions, the stimuli could either be spatially congruent (composing five of the 25 bisensory conditions) or spatially incongruent (composing 20 of the 25 bisensory conditions).

The localization task required subjects to localize all stimuli that were presented on every trial. Specifically, on unisensory auditory trials, subjects had to localize the sound; on unisensory visual trials, subjects had to localize the visual stimulus; on bisensory trials, observers had to localize both the visual *and* the auditory stimulus. Subjects were instructed before the start of the experiment that on bisensory trials, “sometimes the sound and light come from the same location, but sometimes the sound and light come from different localizations.” On these bisensory trials, when the response cursor first appeared, it included a letter inside the cursor that denoted which stimulus the subject should localize first. For half of the subjects, following presentation of bisensory stimuli, the cursor first appeared with a red “S” inside it to indicate the sound was to be localized first; for the other half of subjects, the cursor first appeared with a blue “L” to indicate the light was to be localized first. Following the subjects’ first localization response, the cursor immediately randomized to a new position and displayed the other letter. Thus, on every bisensory trial, we obtained subjects’ estimates for the spatial locations of both the visual and auditory stimuli. While the response order was always the same (e.g., first localize the sound, then light, or vice versa) for a given subject, the order was counterbalanced across subjects, and our previous studies have revealed that response order does not impact localization responses in this task.

Following the “pre-test” session, participants were allowed to take a 10-minute break, and then began the “post-test” session, which employed a “top-up” design with interleaved blocks of an exposure task, and post-test localization trials. The first part of the exposure task lasted for approximately ten minutes, and then would re-appear for brief two-minute sessions every 40 localization trials in the post-test localization phase.

In the exposure task, in experiments #2–#6, a visual flash and auditory burst of sound were presented (on average) every 600 ms. Between every 5th to 15th presentation of the stimuli, the visual flash would get noticeably brighter (from 0.41 to 3.2 cd/m^2^), and participants would have to detect this change in brightness by clicking the mouse. If they correctly detected the change, the stimuli would change spatial positions, with the visual and auditory stimuli switching to opposite sides of the midline.

The *spatial* relationships between the repeated visual and auditory stimuli in the exposure task were as follows: in three experiments (experiments 2, 5, and 6), the visual and auditory stimuli were *incongruent* and presented from spatially discrepant locations (either +19° for vision and −19° for audition, or +19° for audition and −19 for vision, depending on the trial). In two experiments (experiments 3 and 4), they were presented from the same location (either +19° and −19°), and alternated the side they were presented on at the same time to maintain spatial congruency. If participants missed the brightness change, they had to wait through another cycle for a brighter flash to be presented. Participants were forced to maintain fixation at 0°, and if fixation was broken (as detected by the eyetracker), the stimuli paused until fixation at the proper location was regained.

The *temporal* relationships between the repeated visual and auditory stimuli in the exposure task were the following: in two of the experiments (experiment #2 and #4), the temporal stimuli were “uncorrelated” during the exposure task, as the auditory stimulus was drawn from a uniform distribution from −250 ms to +250 ms of when the visual flash was presented. In experiments #3 and #5, the visual and auditory stimuli were “congruent” and were presented synchronously. In experiment #6, the visual stimulus always preceded the auditory stimulus by 400 ms, and thus was perfectly “correlated” with the auditory signal, but was always presented with a fixed temporal discrepancy. (See Table 1 for a summary of the spatial and temporal relationships between stimuli in different experiments).

In the post-test phase, the initial exposure task block lasted for 35 correct detections (approximately ten minutes), and was followed by a localization block consisting of 40 trials. Then, for the remainder of the post-test phase, exposure-task blocks (requiring eight detections) and localization blocks (40 trials) alternated until 525 localization trials were completed. Typically, the post-test phase lasted between 60 and 75 min.

## Results

We fit a Bayesian causal inference model (Odegaard, Wozny & Shams, 2015; Odegaard, Wozny & Shams, 2016) with eight free parameters (see Materials and Methods for details, and the Supplemental Information for the parameter tables) to each individual subjects’ data to acquire an estimate of the binding tendency in the pre-test and post-test localization phases. The average fit for each model across subjects was quite reliable for both the pre-test and the post-test datasets (Pre-Test *R*^2^ Mean = .82, SD = .19; Post-Test *R*^2^ Mean = .81, SD = .24) (Nagelkerke, 1991).

**Figure 4:**
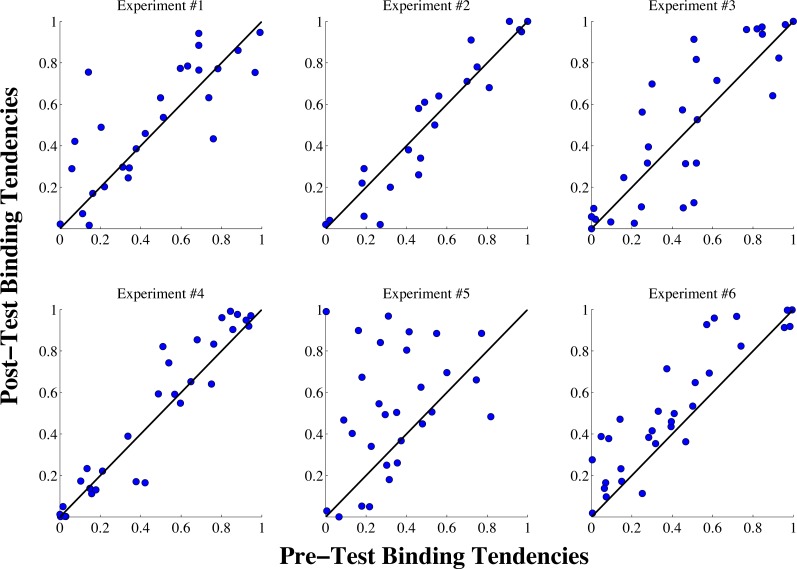
Individual binding tendency scores from the pre-test and post-test sessions for all six experiments. The blue dots show the binding tendency values for each individual subject, and the black lines show the identity line for each plot, indicating perfect stability of the binding tendency from the pre-test to the post-test session. As can be seen in the figure, in experiments #1–4, with the exception of a few outliers, most subjects exhibited consistent binding tendency scores from the pre-test to the post-test sessions. In experiment #5, most subjects displayed an increase in the binding tendency was displayed in the post-test phase, with some heterogeneity among subjects in this trend. Similarly, an increase in the binding tendency was exhibited by most subjects in experiment #6, but the overall trend was smaller than in Experiment #5.

Visualization of changes in the binding tendency on a subject-by-subject basis can be informative to reveal both overall trends and heterogeneity in the potential effects produced by different experiments. Figure 4 displays how the binding tendency for each individual subject changed in each experiment. As can be seen in the figure, with the exception of a few outliers, binding tendency values appear relatively stable across sessions in experiments 1–4. (For a violin plot of this same data showing means and medians of the binding tendency changes for each experiment, see Fig. S1 in the Supplemental Information). The most intriguing trend based on visualizing individual data appears in experiment 5, where a majority (17) of participants displayed an *increase* in the binding tendency from the pre-test to the post-test session. Interestingly, however, 12 of the subjects displayed binding tendency values that are either at or slightly below the pre-test value, indicating there may be heterogeneity in the efficacy of this effect.

To definitively determine whether any significant differences were produced by the exposure tasks in our six experiments, we used a bootstrap sampling approach to evaluate changes in the binding tendency between the pre-test and post-test phases. Our first analysis involved computing 10,000 sample means (with replacement) of the binding tendency differences scores (post test—pre test) for each experiment to construct a 95% confidence interval, and determine whether this confidence interval contained 0, which would indicate that the binding tendency did not significantly change from the pre-test to the post-test phases. To ensure our findings would not be influenced by extreme outliers (as the scatterplots in Fig. 4 reveal a few anomalous subjects), we excluded any data points that fell beyond the value of four times the mean of Cook’s distance for a given experiment.

The values from this initial bootstrapped analysis for our first four experiments were the following: Experiment 1: Mean = .034, 95% CI [−.026, .093]; Experiment 2: Mean = 0, 95% CI [−.043, .042]; Experiment 3: Mean = .037, 95% CI [−.034, .106]; Experiment 4: Mean = .029, 95% CI [−.002, .061]. All bootstrapped confidence intervals for the first four experiments contained 0, indicating the binding tendency did not change across sessions. Even in experiment 4, where the confidence interval barely contained 0, the overall magnitude of the increase was quite small, indicating a negligible effect. Interestingly, however, this same comparison for Experiments 5 and 6 yielded the following results: Experiment 5: Mean = .155, 95% CI [.057, .253]; Experiment 6: Mean =0.113, 95% CI [.065, .162]. This analysis provided initial evidence that the binding tendency changes were considerably higher (and different from 0) in Experiments 5 and 6.

Our second analysis, though, focused on a more rigorous test: comparing the results of Experiments 5 and 6 to the original control experiment. To this end, we sampled (with replacement) the difference scores from Experiments 1, 5 and 6 simultaneously 10,000 times, and on each iteration calculated the mean of the differences between the Experiment 5/6 scores and the control scores. We then computed means and confidence intervals on these distributions, which yielded the following results: Experiment 5 vs. Experiment 1: Mean = .12, 95% CI [0.012, .26]; Experiment 6 vs. Experiment 1: Mean = .079, 95% CI [.005, .152]. For Experiment 5, the confidence interval did not contain 0, indicating a significant increase in Experiment 5 when compared to the control. This was also true of experiment 6, but by a smaller margin; the lower end of the confidence interval was almost exactly 0.

Interestingly, none of the other experiments differed significantly from the control experiment. This same analysis for the other three experiments yielded the following results: Experiment 2 vs. Experiment 1: Mean = −.033, 95% CI [−.099, .032], Experiment 3 vs. Experiment 1: Mean =0.003, 95% CI [−0.085, 0.092], Experiment 4 vs. Experiment 1: Mean = −.005, 95% CI [−.068, .059]. Therefore, while Experiment 5 and Experiment 6 provide evidence for increases in the binding tendency from the pre-test to the post-test phase, none of the other experiments revealed a change in this parameter. All of these results are summarized in Table 2.

**Table 2:** Summary of experimental changes in the binding tendency parameter, based on the temporal and spatial characteristics of the exposure-task stimuli in different experiments.

Experiment	Time	Space	Binding tendency: actual change
1	None	None	None
2	Uncorrelated	Incongruent	None
3	Congruent	Congruent	None
4	Uncorrelated	Congruent	None
5	Congruent	Incongruent	Increase
6	Correlated	Incongruent	Marginal increase

**Figure 5:**
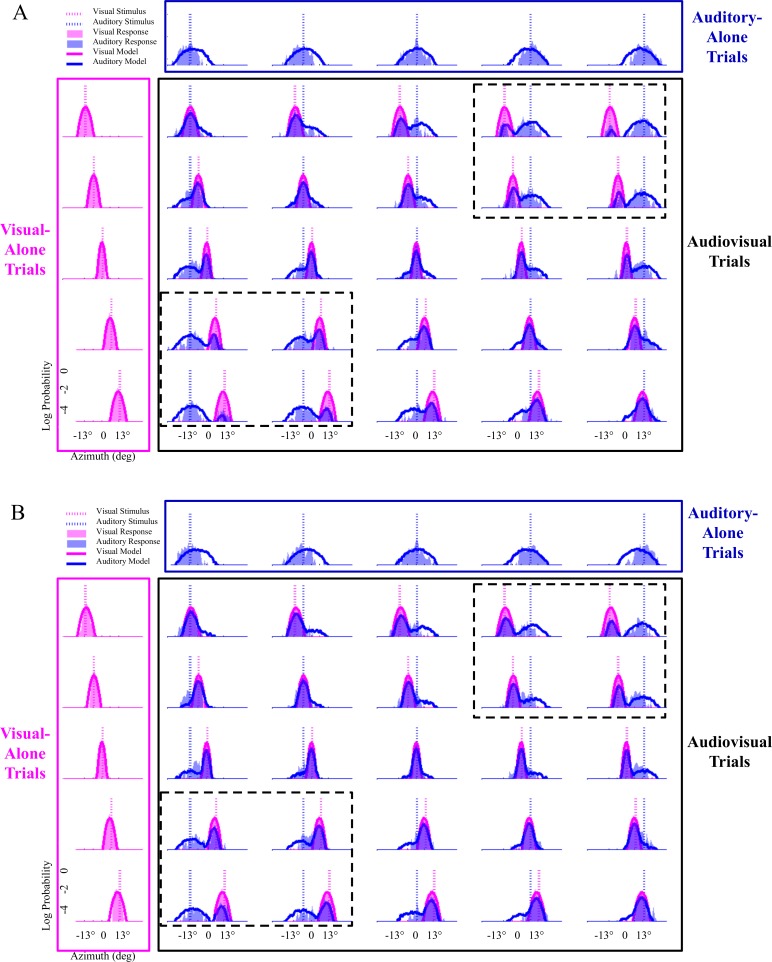
Modeling results with data from all subjects in experiment #5 that were best characterized by the probability-matching strategy. (A) Pre-test localization data from twenty subjects are shown, with the model fit to all of these subjects’ data at once. Visual-only conditions are shown in the pink boxes, auditory-only conditions in the dark blue boxes, and audiovisual conditions in the black boxes. Audiovisual trials with stimuli played at the same location are along the diagonal in the black box. The bisensory conditions with the highest amount of spatial discrepancy between the visual and auditory stimuli are shown in the black boxes marked by the dotted lines in each figure. (B) Post-test localization data. The conditions in the black boxes with dotted lines clearly reveal the increase in binding tendency from the pre-test to the post-test phase, as the amount of overlap between the pink (visual) and blue (auditory) response distributions increases from the pre-test to the post-test phase, showing that a larger percentage of auditory stimuli were localized where the visual stimuli occurred on these spatially-discrepant trials.

The increase in the binding tendency in Experiment 5 can be seen in the change in many subjects’ behavioral data and model fits from the pre-test and post-test phases. Shown in Fig. 5 is all of the data from subjects that were best fit by the probability-matching strategy in our model, as well as the modeling results when fit to this aggregate dataset. In the conditions with spatially discrepant audiovisual stimuli, the increase in the overlap between the visual and auditory response demonstrates the increase in tendency to integrate spatially discrepant visual and auditory signals.

## Discussion

One of the important questions in a Bayesian account of perception is how priors can change over time as new sensory experience is acquired (Seriès & Seitz, 2013). While previous studies have shown that priors can be modified by sensory experience (e.g., Chalk, Seitz & Seriès, 2010; Adams, Kerrigan & Graf, 2010; Sotiropoulos, Seitz & Seriès, 2011), and that associations between arbitrarily paired signals across modalities can be learned (Ernst, 2007; Shams et al., 2011; Altieri et al., 2015), it is not currently clear to what degree brief sensory experiences cause the audiovisual binding tendency to change, and what the conditions are that result in an update of this mechanism the brain. Our results provide preliminary evidence that the prior tendency to bind/integrate audiovisual signals *can* change substantially due to brief sensory experiences, and that this tendency seems to increase after repeated exposure to spatially *discrepant* audiovisual stimuli that are presented with fixed temporal relationships, but not after exposure to spatiotemporally concordant stimuli. Specifically, repeated exposure to auditory and visual stimuli that were mismatched in space (in a variable fashion, reversing positions frequently, Fig. 1B) but had a synchronous temporal relationship resulted in an increase in the tendency to bind on localization trials in the post-test phase.

The result from experiments 5 (and strong trend in experiment 6) seems consistent with the framework of our second hypothesis (see Figs. 2B and 2D). We note that both of the original hypotheses discussed in this paper can be interpreted as reflecting error correction mechanisms, but operating on *different kinds of errors*. To clarify the distinction between these two types of errors and clarify the hypotheses motivated by each construct, we focus on Experiment 5 (which contained our main result) and contrast the predictions of the two hypotheses for this specific experiment.

### Explanation of the original hypotheses about how priors are updated

Regarding our first hypothesis (Figs. 2A and 2C), let us assume that the observer starts the experiment with an *a priori* binding tendency of .25, which would predict that, on average, flashes and beeps have a common cause 25% of the time. During the Exposure phase, the observer is exposed to many trials with high spatial discrepancy. If 95% of these trials result in an inference of independent causes, then the observer experiences a 5% frequency of common cause during this phase which is at odds with the *a priori* expectation of 25%. According to this hypothesis, this should cause a “correction” of binding tendency by reducing it. In this framework, the nervous system treats its inference about spatially discrepant sensory signals as ground truth, and adjusts its prior accordingly to match the statistics of the world.

On the other hand, according to our second hypothesis, the brain arguably has a model of the world in which sensations with highly discrepant spatial locations generally have independent causes, and the degree of “tolerance” for the discrepancy is determined by the *a priori* expectation of a common cause (or binding tendency). The larger the binding tendency, the higher the tolerance to discrepancy in signals for inferring a common cause. Now, given a binding tendency of .25, highly discrepant auditory and visual signals (e.g., of 39 degrees apart) are inferred to have independent causes on most trials. However, the observance of repeated temporal coincidence across trials provides independent and strong evidence of a common cause. Given the good temporal precision in both modalities and poor spatial precision for auditory signals, the repeated temporal coincidence provides an evidence of common cause that overrides the spatial discrepancy (which is less reliable) and results in an interpretation of common cause for these stimuli after repeated exposure. This inference contradicts the model of the world (“highly discrepant signals have independent causes”) and leads to “correction” of the model by increasing the binding tendency.

Therefore, while both of our hypotheses rely on statistics of the observed stimuli, because they make different assumptions about how the causal inference proceeds across trials, they make opposite predictions for learning. The results from Experiment 5 could be interpreted to be consistent with the predictions of the a “predictive coding” mechanism in the brain (Rao & Ballard, 1999; Friston, 2003; Friston, 2005; Friston, 2012; Friston & Kiebel, 2009). That is, because the apparent mechanism that updates the prior relies on production of an error between model prediction and sensory observation, it could be considered to be compatible with previous ideas about how predictive coding may be implemented.

We can only speculate why the mechanism specified by our second hypothesis appears to operate, rather than the mechanism specified by the first hypothesis; we posit that the correction (or updating) of the binding tendency, which is a prior, cannot depend on constructs that depend on that prior. To correct a variable, information may need to come from a source which is independent from that variable; that is, to correct this prior, the information can come from sensory observations, but not from an inference that is derived from that prior. In the scenario specified by the first hypothesis, the inference of independent causes on each trial can only occur based on a low binding tendency; it cannot be entirely based on the stimuli themselves, because while the stimuli are spatially discrepant, they are temporally consistent. Therefore, an inference which is itself based on the binding tendency cannot be a reliable source for updating the binding tendency. Whereas under the second hypothesis, the evidence of a common cause is merely based on the sensory observations; namely, the repeated temporal coincidence (and the low spatial resolution of auditory signals). Therefore, this is a source of information that is independent of the prior (binding tendency), and thus, can serve as the basis to correct the prior.

This remodeling of the brain’s internal model of the world appears to occur because the repeated co-occurrence of otherwise unrelated/discrepant stimuli is treated by the nervous system as compelling evidence of unity or association between the signals (Figs. 2B and 2D). Unless the nervous system relaxes its criterion for perception of unity, it cannot account for the repeated co-occurrence of the stimuli. This modification of the prior in order to better account for compelling sensory regularity has been observed in other phenomena (Chalk, Seitz & Seriès, 2010; Sotiropoulos, Seitz & Seriès, 2011), and one study has referred to it as the “oops” factor (Adams, Kerrigan & Graf, 2010). While it is possible that the mechanism characterized by hypothesis #1 may play a relevant role on each trial in incrementally updating multisensory priors, it appears that here, the modification of the priors is due to large error signals, and thus the mechanism specified by hypothesis #2 likely underlies this substantial change in the prior for binding sensory signals across space.

### Relating the present results to previous empirical findings

Considering our main empirical findings, it is important to comment on how our results relate to previous research on audiovisual recalibration. Previous studies have shown that repeated exposure to auditory and visual stimuli with fixed spatial discrepancy results in a modification of auditory spatial perception (Canon, 1970; Radeau & Bertelson, 1977; Recanzone, 1998; Lewald, 2002; Frissen et al., 2003; Frissen et al., 2005; Kopco et al., 2009; Eramudugolla et al., 2011). This phenomenon is known as the “ventriloquist aftereffect” and has been computationally accounted for by a shift in the auditory sensory representations (likelihood functions) rather than a change in spatial prior expectations (Wozny & Shams, 2011b). Here, for the first time, we report that repeated exposure to auditory-visual stimuli with *variable* spatial discrepancy (i.e., discrepancy which alternated between +39° and −39°) results in a modification of the binding tendency. Therefore, it appears that repeated exposure to spatially discrepant auditory-visual stimuli always results in an update in perceptual processing, with the type of the update depending on relationship between the adapting stimuli. If the spatial relationship in the adapting stimuli is fixed, the nervous system appears to treat the more precise signal as the “teaching” signal and recalibrate the less precise representations by shifting them to reduce the discrepancy. If the relationship is variable, the sensory discrepancy cannot be rectified by a simple shift in sensory representations, and the nervous system does not try to “correct” either sensory representation. Instead, it attempts to “correct” its model of the world by relaxing its criterion for the perception of unity.

It is also important to note the relationship between our present results and previous findings in the literature regarding how sensory experiences can influence integration on subsequent encounters. Recent studies have primarily investigated this question in the context of multisensory speech perception (Nahorna, Berthommier & Schwartz, 2012; Nahorna, Berthommier & Schwartz, 2015; Gau & Noppeney, 2016). These results show that prior exposure to asynchronous or incongruent audiovisual speech combinations decrease participants’ tendency to perceive the McGurk illusion. At first glance, these findings may appear to contradict the results reported here, but a closer look at these studies reveals important differences in the stimuli and paradigms. For instance, in the strongly incongruent condition in Nahorna, Berthommier & Schwartz (2015), there is no temporal consistency between the auditory and visual modalities that could potentially provide evidence of a common cause; in the phonetically incongruent conditions in studies by Nahorna, Berthommier & Schwartz (2012) and Nahorna, Berthommier & Schwartz (2015) and the incongruent condition in Gau & Noppeney (2016), which are more similar to incongruent condition in our study, the “incongruent” speech stimuli used syllables that are *never* integrated to produce the McGurk illusion. It is possible that if the stimuli are along independent or orthogonal dimensions, then exposure to this type of completely unrelated stimuli, which *cannot possibly* be integrated, may activate the mechanism from our first hypothesis and cause a decrease in the tendency to integrate. In contrast, in our study, the sounds and sights are along a dimension that brain can integrate over, depending on the binding tendency. Therefore, modification of binding tendency in this scenario *can* reduce the prediction error, and the mechanism from our second hypothesis can operate.

Another study relevant for our present results is by Van Wanrooij, Bremen & Van Opstal (2010), which investigated the effects of frequency of auditory-visual spatial alignment on multisensory spatial integration. In this study, integration was measured primarily by reaction times, and the main finding was that spatially aligned audiovisual stimuli elicited the fastest reaction times to targets (i.e., the visual component of the AV stimuli) in blocks when the stimuli were *always* aligned (100–0 condition) compared to blocks with 50–50 or 10–90 ratios for AV alignment. While these results suggest that the binding tendency in the 100–0 condition was higher than that in the other two conditions, we are not certain if this difference can be entirely attributed to the statistics of and exposure to the stimuli in the blocks, since the instructions given to subjects in the three conditions differed: in the 100-0 condition, subjects were instructed that the audiovisual stimuli were presented in spatiotemporal alignment and that the flash was the target (pp. 1764–1765), while in the other conditions, subjects were told to ignore the auditory stimuli, and the methods do not indicate that subjects had any *a priori* knowledge about the stimulus distributions.

We think it is critical that future studies include baseline measures of integration before evaluating how context plays a role, and include plots to show how individual data points may be impacted by context and exposure. As has been recently noted, using bar graphs alone can conceal important trends in data (Rousselet, Foxe & Bolam, 2016); thus, we strongly recommend that future studies plot individual data points to make the effects of context and exposure on integration more interpretable. We also acknowledge it is possible that there are factors that are at work that we are not considering that could explain these differences in findings. To ascertain what factors drive changes in binding tendency, and what the underlying mechanisms are, future studies will need to specify a taxonomization of influences on perceptual priors by noting how tasks, stimuli, instructions, and other factors can impact this mechanism.

We consider this study to be one piece of evidence to demonstrate that the binding tendency can change quickly, but additional research is needed to catalogue the wide array of possible influences on binding tendency. We think that the binding tendency (as with most other priors) is likely formed and influenced by both “low-level” and “high-level” factors. The low-level factors would include prior experience of the relationship between certain sounds and sights. This factor is influenced by the stimulus statistics and regularities and does not involve top-down decisional factors *per se*. On the other hand, higher-level factors such as explicit cognitive knowledge about the stimuli (e.g., knowledge obtained through instructions given by the experimenter) can also influence the expectation of a common cause and hence, the binding tendency. Many perceptual learning studies have shown that perceptual learning can occur without feedback; therefore, we do not find it surprising that an update of the binding tendency can occur implicitly and without feedback.

### Future directions for studying the binding tendency

The current findings demonstrate that this plastic capacity of the brain can occur in an extremely short period of time (less than ten minutes), revealing an adaptive ability of the system to learn about new associations between sensory signals in the environment, even in adulthood. Considering that deficits in sensory integration are thought to accompany several disorders, including dyslexia (Hairston et al., 2005; Harrar et al., 2014; Hahn, Foxe & Molholm, 2014), autism (Stevenson et al., 2015; Stevenson et al., 2014a; Stevenson et al., 2014b; Wallace & Stevenson, 2014; Baum, Stevenson & Wallace, 2015), and schizophrenia (Ross et al., 2007; Szycik et al., 2009; Williams et al., 2010; Zvyagintsev et al., 2013; Stekelenburg et al., 2013), by illuminating basic principles underlying how the tendency to bind sensory signals across space can be modified, we think these findings represent a key contribution as the field attempts to develop effective remedial or educational protocols for enhancing multisensory integration in clinical populations (Wallace & Stevenson, 2014).

For instance, it has recently been shown that an auditory training protocol can improve higher-level cognitive functioning in young individuals with early-onset schizophrenia (Fisher et al., 2015). Indeed, recent data from our laboratory indicates that individuals with low binding tendency scores on a spatial localization task report higher numbers of psychometrically-defined prodromal features in their everyday lives (Odegaard & Shams, in press). Thus, if a protocol can be developed to improve lower-level sensory binding in individuals with schizophrenia, it seems possible that downstream, higher-level functions could also improve. Currently, we can only speculate whether increasing the binding tendency can ameliorate symptoms in clinical disorders, but we think recent results demonstrating increased executive functioning with sensory training protocols in individuals with schizophrenia are promising (Fisher et al., 2015; Dale et al., 2016), and that our findings can help inform future investigations looking to exploit multisensory techniques to modify how audiovisual information is integrated in the brain.

### Limitations of this investigation

Finally, while the six experiments presented here represent an important, first demonstration of audiovisual binding tendency modification, it is important to note limitations of the current study and what is currently unknown regarding modification of this mechanism in the brain. First, future studies will be needed to uncover further specifics about the phenomenon, including how the increase in the spatial binding tendency may scale with the spatial discrepancy between stimuli presented in the exposure task, how long the increase in binding may last following exposure, and whether the principles reported here generalize to other domains and stimuli. For example, would a 10-minute exposure to McGurk speech stimuli, which are consistent in time but discrepant in syllabic content, increase the tendency to bind audiovisual speech signals? Considering the current findings, this is an intriguing hypothesis. The question of how exposure to stimuli that have a fixed temporal relationship but *vary* in terms of the magnitude of spatial discrepancy also remains unanswered. We posit that this type of condition would still cause a slight increase in the tendency to bind; indeed, one may interpret the slight increase in binding tendency in our control experiment in support of this hypothesis, as exposure to our localization task (with 20 out of 35 conditions containing spatially discrepant stimuli) appears to slightly increase the tendency to bind, but this question will need to be addressed more thoroughly by future work.

Second, we recently demonstrated that the brain’s tendency to bind auditory and visual sensory signals does not generalize across tasks (Odegaard & Shams, 2016). Therefore, we do not expect that the changes in binding tendency observed in Experiment 5 and 6 would generalize to temporal tasks. However, the question of how general the changes in binding tendency induced in this fashion are, and to what other stimuli, tasks, perceptual domains and contexts they would generalize to is a very important question, both from the perspective of underlying mechanisms and the perspective of translation to clinical and educational applications. Another open question is whether the same type of plasticity would operate in the temporal domain; namely, whether repeated exposure to sensory signals that are temporally discrepant but spatially congruent increase the tendency to bind sensory signals in a temporal task, More broadly, the question of which spatial and temporal relationships between audiovisual stimuli in the exposure task would cause an update in the temporal binding tendency in the brain. These important questions remain unanswered, and will need to be investigated by future studies.

Third, the fact that none of our experiments resulted in a *decrease* in the binding tendency is also interesting, and the conditions that cause a *reduction* in the tendency to integrate audiovisual information across space remain unknown. It remains possible that a protocol in which there is repeated congruency in temporal and spatial dimensions, but compelling evidence that the signals originate from different sources (thus creating a mismatch between model predictions and sensory experience) would lead to a decrease in binding tendency. Also, recent studies have provided preliminary evidence that active forms of sensory training (for example, training subjects to discriminate audiovisual signals with greater precision) can decrease the tendency to bind (McGovern et al., 2016). This contrasts our results here, which are based on a more passive form of exposure. Therefore, future studies should further examine the conditions in which the binding tendency may decrease by probing whether semantic incongruency, attentional factors, or other indicators that the signals originate from separate sources are necessary to induce this type of change.

Lastly, the neural mechanism underlying the binding tendency currently remains unknown, and additional work will be needed to uncover how this tendency is encoded in the brain. Recent work using functional magnetic resonance imaging reveals some initial insight into how the binding tendency might be encoded in the brain (Rohe & Noppeney, 2015a), but work using additional methods, such as neural network modeling, will be necessary to uncover the specific neural mechanisms (e.g., the specific type of connectivity, neural architecture, circuitry and physiology) that determine binding tendency in the brain.

To conclude, we think these findings from our large dataset from six experiments represent a pivotal first step in learning about how the binding tendency can be modified, and we hope future investigations will build upon these results in answering many questions that remain as the study of multisensory processing continues to investigate both translational and clinical applications of this work.

##  Supplemental Information

10.7717/peerj.3143/supp-1 Supplemental Information 1 Supplemental information The supplemental information contains two additional figures and the tables displaying the mean & SEM of the optimized parameter fits for each experiment.

10.7717/peerj.3143/supp-2 Supplemental Information 2 Explanation of parameter tables The explanation of the parameter tables for each experiment.

10.7717/peerj.3143/supp-3 Supplemental Information 3 Optimized parameters for all experiments The optimized parameters for each subject for each experiment.

10.7717/peerj.3143/supp-4 Supplemental Information 4 Explanation of behavioral data The explanation of the behavioral data for each experiment.

10.7717/peerj.3143/supp-5 Supplemental Information 5 Raw data The behavioral data for each experiment.

10.7717/peerj.3143/supp-6 Supplemental Information 6 Sample Bayesian causal inference model A sample modeling file to implement the Bayesian causal inference model described in this paper. Please note that versions that implement the analytic solutions will run much faster, but this file should make the relevant computations more explicit.

## Additional Information and Declarations

### Data Availability

The following information was supplied regarding data availability: The raw data has been supplied as a Supplementary File.
