# Supplementary material for: A simple and efficient method to enhance audiovisual binding tendencies
Source: PeerJ. 2017 Apr 25;5:e3143. doi: 10.7717/peerj.3143 (PMC5407282; doi:10.7717/peerj.3143)
Supplement: Supplemental Information 1 — The supplemental information contains two additional figures and the tables displaying the mean & SEM of the optimized parameter fits for each experiment. [file peerj-05-3143-s001.docx]

**Supplemental Information**

Figure S1 shows the distributions of the differences in binding tendency scores (post-test – pre-test) for all six experiments. As can be seen in the plot, Experiments 1-4 exhibited relatively small differences in the average binding tendency between sessions. The exposure conditions that produced the largest changes in the binding tendency were Experiments 5 and 6 in which the visual and auditory exposure stimuli were spatially separated by ±39 degrees (alternating their spatial positions during the exposure task, Figure 1B) and presented with a fixed temporal relationship (stimuli were presented either simultaneously or consistently staggered by 400ms). These exposures resulted in a substantial amount of increase, with an average increase of 18% in Experiment 5 and 11% in Experiment 6, despite the brief duration of exposure. (To see the behavioral correlate of these changes in the binding tendency, please see Supplementary Figure S2.)
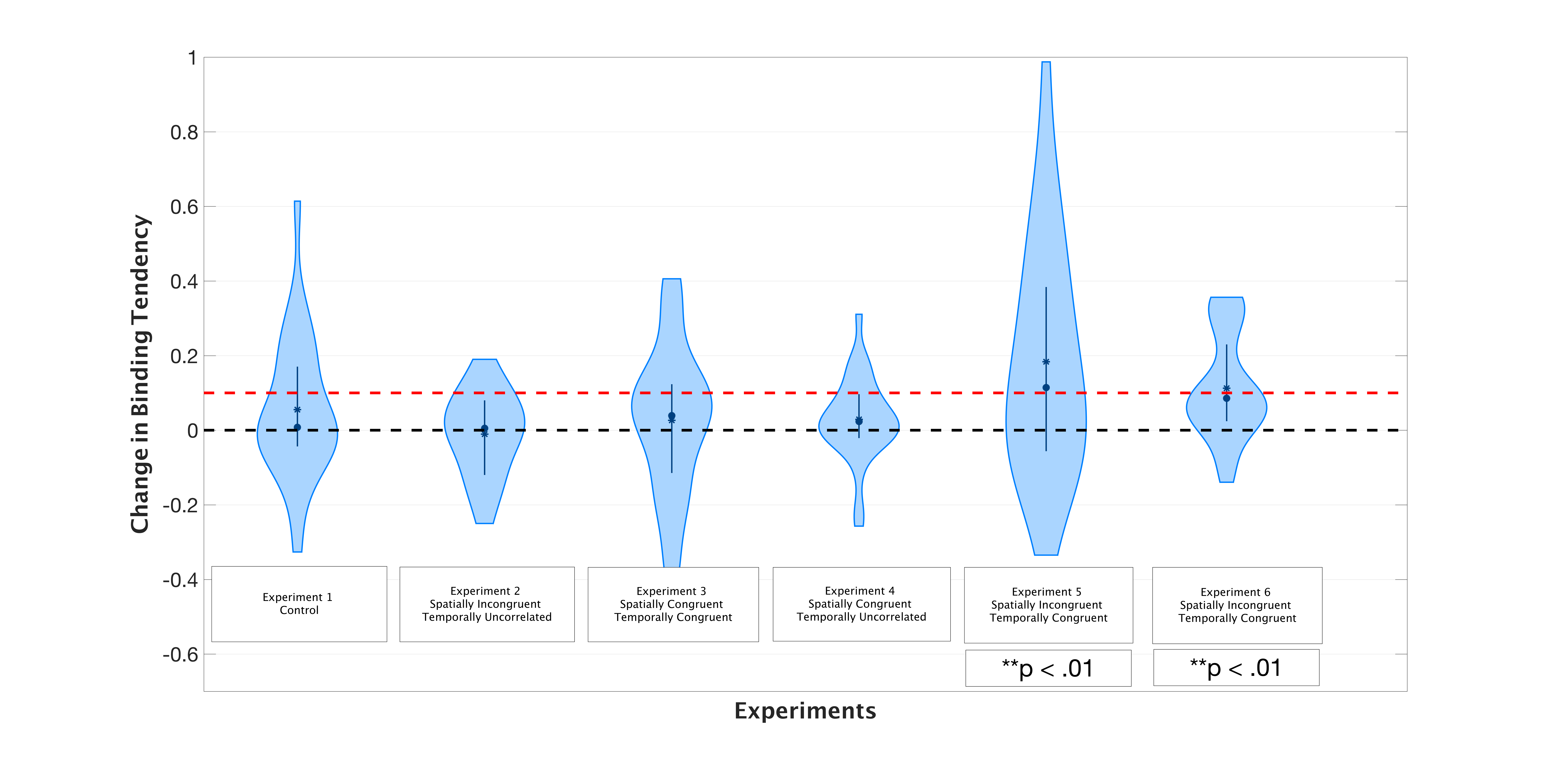


Figure S1. The changes in binding tendencies from the pre-test to the post-test localization phase for each experiment. This violin plot displays the distribution of binding tendency difference scores (post-test – pre-test) for all six experiments. For each experiment, the solid circle represents the median change, the star represents the mean change, and the solid line is the inter-quartile range. The width of each violin plot represents the distribution of the individual difference scores. The dotted black line represents no change in the binding tendency, while the red dotted line reflects a 10% increase in the binding tendency. Significance was determined by Wilcoxon signed-rank tests, comparing pre-test and post-test binding tendency values; please note that the determination of significance in the main manuscript involved a bootstrap sampling approach, but both of these analyses yielded the same conclusions.

**Behavioral Results from Experiments 1-6**

**

**

Figure S2. Auditory bias as a measure of auditory-visual interactions. The average auditory bias (as computed in Kording et al., 2007: A_response_ – A_location_/V_location_ - A_location_), across all twenty spatially-discrepant conditions in pre-test and post-test phases is shown for each Experiment. Errors bars indicate SEM. The difference between average auditory bias in the pre-test and the post-test phase was significant in Experiment 5 (Wilcoxon signed rank test: z = 2.77, p < .01). There was also a trend for increase in bias in Experiment 6; however, this trend was not statistically significant using this nonparametric test (Wilcoxon signed rank test: z = 0.63, p = 0.53). Examination of model parameters for Experiment 6 (see Table 6), suggests that the effect of increase in binding tendency is somewhat offset by a small increase in auditory precision in the post-test phase, reducing the overall degree of average auditory bias.

**Additional Results**

**Average Optimized Parameter Values for the Best-Fitting Models**

*Experiment #1 (N = 27)*

|  | Prior Parameters | | | Likelihood Parameters | | | | |
| --- | --- | --- | --- | --- | --- | --- | --- | --- |
|  | B_t_ | σ_p_ | x_p_ | σ_v_ | Δσ_v_ | Δx_v_ | σ_a_ | Δx_a_ |
| Pre-Test | 0.46  (± .06) | 21.57  (± 2.79) | -0.07  (± 1.71) | 1.38  (± .12) | 0.44  (± .05) | -0.64  (± .08) | 15.37  (± 1.76) | 4.58  (± .87) |
| Post-Test | 0.51  (± .06) | 21.26  (± 3.38) | 2.37  (± 1.41) | 1.50  (± .13) | 0.45  (± .04) | -0.67  (± .08) | 17.35  (± 2.09) | 5.61  (± .82) |

**Table 1.** Optimized Parameter values ± standard error parameter estimates from experiment 1. None of the parameters changed significantly from the pre-test to the post-test localization session (p > .05 for all Wilcoxon signed rank tests).

*Experiment #2 (N = 22)*

|  | Prior Parameters | | | Likelihood Parameters | | | | |
| --- | --- | --- | --- | --- | --- | --- | --- | --- |
|  | B_t_ | σ_p_ | x_p_ | σ_v_ | Δσ_v_ | Δx_v_^*^ | σ_a_^*^ | Δx_a_ |
| Pre-Test | 0.52  (± .07) | 24.64  (± 4.23) | 0.69  (± 2.14) | 1.40  (± .13) | 0.59  (± .06) | -0.32  (± .12) | 13.38 (± 1.50) | 2.71  (± .86) |
| Post-Test | 0.51  (± .07) | 25.62  (± 4.07) | 1.60  (± 2.02) | 1.70 (± .20) | 0.49  (± .03) | -0.61  (± .14) | 16.66 (± 1.54) | 1.59  (± 1.0) |

**Table 2**. Optimized parameter values ± standard error parameter estimates from experiment 2. The only parameters that changed significantly from the pre-test to the post-test localization session were the visual likelihood delta parameter, which exhibited a slight change of a few tenths (Wilcoxon signed rank test: z = 3.52, p < .0001), and a slight increase in the auditory likelihood parameter (z = 2.52, p = 0.01).

*Experiment #3 (N = 29)*

|  | Prior Parameters | | | Likelihood Parameters | | | | |
| --- | --- | --- | --- | --- | --- | --- | --- | --- |
|  | B_t_ | σ_p_ | x_p_ | σ_v_ | Δσ_v_ | Δx_v_ | σ_a_ | Δx_a_ |
| Pre-Test | 0.47  (± .06) | 20.75  (± 3.36) | 0.52  (± 1.29) | 1.17  (± .10) | 0.64  (± .09) | -0.70  (± .12) | 18.39  (± 1.82) | 4.97  (± .75) |
| Post-Test | 0.49  (± .07) | 26.97  (± 4.31) | 0.64  (± 1.39) | 1.18  (± .13) | 0.57  (± .07) | -0.74  (± .12) | 17.05  (± 1.82) | 4.35  (± 1.02) |

Table 3. Optimized parameter values ± standard error parameter estimates from experiment 3. None of the parameters changed significantly from the pre-test to the post-test localization session (p > .05 for all Wilcoxon signed rank tests).

*Experiment #4 (N = 30)*

|  | Prior Parameters | | | Likelihood Parameters | | | | |
| --- | --- | --- | --- | --- | --- | --- | --- | --- |
|  | B_t_ | σ_p_ | x_p_ | σ_v_ | Δσ_v_ | Δx_v_^*^ | σ_a_ | Δx_a_ |
| Pre-Test | 0.46  (± .06) | 29.68  (± 4.95) | 1.42  (± 1.14) | 1.33  (± .11) | 0.58  (± .04) | 0.16  (± .08) | 18.03  (± 1.73) | 5.81  (± .73) |
| Post-Test | 0.49  (± .07) | 30.19  (± 5.25) | 1.06  (± 1.07) | 1.40  (± .11) | 0.54  (± .04) | -0.04  (± .08) | 16.95  (± 1.79) | 5.38  (± .72) |

**Table 4.** Optimized parameter values ± standard error parameter estimates from experiment 4. The only parameter that changed significantly from the pre-test to the post-test localization session was a slight change in the visual likelihood delta parameter (Wilcoxon signed rank test: z = 3.12, p = .002).

*Experiment #5 (N = 29)*

|  | Prior Parameters | | | Likelihood Parameters | | | | |
| --- | --- | --- | --- | --- | --- | --- | --- | --- |
|  | B_t_^*^ | σ_p_ | x_p_ | σ_v_ | Δσ_v_ | Δx_v_ | σ_a_ | Δx_a_ |
| Pre-Test | 0.34  (± .04) | 19.76  (± 2.93) | -1.30  (± 1.23) | 1.20  (± .08) | 0.51  (± .05) | -0.44  (± .08) | 15.88  (± 1.73) | 4.61  (± 0.90) |
| Post-Test | 0.52  (± .06) | 24.20  (± 4.20) | -0.70  (± 1.22) | 1.23  (± .09) | 0.47  (± .05) | -0.61  (± .08) | 17.28  (± 1.76) | 2.95  (± 0.86) |

**Table 5.** Optimized parameter values ± standard error parameter estimates from experiment 5. The binding tendency changed significantly from the pre-test to the post-test (Wilcoxon signed rank test: z = 2.64, p = .008), and there was also a small but significant change in the visual likelihood delta parameter (Wilcoxon signed rank test: z = 3.17, p = .002). Please note, however, that the main comparison for the binding tendency involves a bootstrapped analysis, which we outline in the main manuscript.

*Experiment #6 (N = 31)*

|  | Prior Parameters | | | Likelihood Parameters | | | | |
| --- | --- | --- | --- | --- | --- | --- | --- | --- |
|  | B_t_^*^ | σ_p_ | x_p_ | σ_v_ | Δσ_v_ | Δx_v_^*^ | σ_a_ | Δx_a_ |
| Pre-Test | 0.40  (± .05) | 20.05  (± 3.53) | 2.31  (± 1.14) | 1.20  (± .10) | 0.49  (± .04) | -0.46  (± .10) | 15.53  (± 1.72) | 4.97  (± 0.79) |
| Post-Test | 0.51  (± .05) | 26.28  (± 5.17) | 1.54  (± 1.12) | 1.12  (± .10) | 0.52  (± .04) | -0.66  (± .12) | 14.26  (± 1.62) | 4.04  (± 0.96) |

**Table 6**. Optimized parameter values ± standard error parameter estimates from experiment 6. The binding tendency changed significantly from the pre-test to the post-test (Wilcoxon signed rank test: z = 3.68, p < .0001), and there was also a small but significant change in the visual likelihood delta parameter (Wilcoxon signed rank test: z = 2.96, p = .003). Please note, however, that the main comparison for the binding tendency involves a bootstrapped analysis, which we outline in the main manuscript.
