# Supplementary material for: A simple and efficient method to enhance audiovisual binding tendencies
Source: PeerJ. 2017 Apr 25;5:e3143. doi: 10.7717/peerj.3143 (PMC5407282; doi:10.7717/peerj.3143)
Supplement: Supplemental Information 2 — The explanation of the parameter tables for each experiment. [file peerj-05-3143-s002.docx]

PeerJ Parameter Tables for Odegaard, Wozny, & Shams (2017)

The optimized parameters for all six experiments can be found in the PeerJParameterTables.mat file.

Each row in each matrix represents the optimized parameter set for one subject for an experiment.

The columns in each matrix represent the following:

Column 1: Pre-test phase: Binding Tendency (a.k.a. “pcommon”)

Column 2: Pre-test phase: Visual Likelihood Variance

Column 3: Pre-test phase: Auditory Likelihood Variance

Column 4: Pre-test phase: Visual Likelihood Mean Delta Term

Column 5: Pre-test phase: Auditory Likelihood Mean Delta Term

Column 6: Pre-test phase: Visual Likelihood Variance Delta Term

Column 7: Pre-test phase: Spatial Prior Mean

Column 8: Pre-test phase: Spatial Prior Variance

Column 9: Post-test phase: Binding Tendency (a.k.a. “pcommon”)

Column 10: Post-test phase: Visual Likelihood Variance

Column 11: Post-test phase: Auditory Likelihood Variance

Column 12: Post-test phase: Visual Likelihood Mean Delta Term

Column 13: Post-test phase: Auditory Likelihood Mean Delta Term

Column 14: Post-test phase: Visual Likelihood Variance Delta Term

Column 15: Post-test phase: Spatial Prior Mean

Column 16: Post-test phase: Spatial Prior Variance

These parameter tables can all be found at the end of the Supplemental Information document.
