# Supplementary material for: A simple and efficient method to enhance audiovisual binding tendencies
Source: PeerJ. 2017 Apr 25;5:e3143. doi: 10.7717/peerj.3143 (PMC5407282; doi:10.7717/peerj.3143)
Supplement: Supplemental Information 4 — The explanation of the behavioral data for each experiment. [file peerj-05-3143-s004.docx]

PeerJ Data for Odegaard, Wozny, & Shams (2017)

The data files for this investigation reflect the 1050 localization trials completed by each subject.

“firsthalf” reflects the Pre-Test data, and contains 525 trials.

“secondhalf” reflect the Post-Test data, and contains 525 trials.

“trialData” reflects a vertical concatenation of first half and second half data.

Each row in each .mat file reflects a single trial.

The columns in each .mat file reflect the following:

Column 1: Visual stimulus location

- This is based on the five possible positions used in this study
- From left to right, these positions are labeled -2, -1, 0, 1, and 2
- NaN denotes a trial where a visual stimulus was not displayed

Column 2: Visual stimulus position on the screen in pixels

Column 3: Visual stimulus position on the screen in cm

Column 4: Visual stimulus position on the screen in visual degrees

Column 5: Subject response about where the visual stimulus was in pixels

Column 6: Subject response about where the visual stimulus was in cm

Column 7: Subject response about where the visual stimulus was in visual degrees

Column 8: Subject reaction time to localize the visual stimulus

Column 9: Auditory stimulus location

- This is based on the five possible positions used in this study
- From left to right, these positions are labeled -2, -1, 0, 1, and 2
- NaN denotes a trial where an auditory stimulus was not displayed

Column 10: Auditory stimulus position (from just behind the screen) in pixels

Column 11: Auditory stimulus position (from just behind the screen) in cm

Column 12: Auditory stimulus position (from just behind the screen) in visual degrees

Column 13: Subject response about where the auditory stimulus was in pixels

Column 14: Subject response about where the auditory stimulus was in cm

Column 15: Subject response about where the auditory stimulus was in visual degrees

Column 16: Subject reaction time to localize the auditory stimulus
